# Usefulness of Noninvasive Hemoglobin Measurement in Community-Dwelling Elderly People

**DOI:** 10.7759/cureus.91626

**Published:** 2025-09-04

**Authors:** Yuta Sato, Tadayuki Iida, Satomi Aoi, Ruriko Miyashita, Hiromi Ikeda, Yoko Okuyama, Keiko Kanagawa, Anri Murakami, Wakako Tsumiyama

**Affiliations:** 1 Physical Therapy, Department of Health and Welfare, Faculty of Health and Welfare, Prefectural University of Hiroshima, Mihara, JPN; 2 Nursing, Department of Health and Welfare, Faculty of Health and Welfare, Prefectural University of Hiroshima, Mihara, JPN; 3 Department of Nursing Women's Health Care, Kobe City College of Nursing, Kobe, JPN; 4 Midwifery, Department of Health and Welfare, Faculty of Health and Welfare, Prefectural University of Hiroshima, Mihara, JPN

**Keywords:** anemia, blood sampling, elderly people, frailty, hemoglobin, noninvasive measurement, respiratory function

## Abstract

Introduction

The evaluation of anemia in elderly people is important; however, routine assessment using traditional blood sampling can be difficult due to its invasiveness and practical constraints. Recently, Rad-67^TM^ (Masimo Corp., Irvine, CA) has become available, which can measure Hb levels noninvasively using a sensor that measures absorbance, such as a SpO2 meter. However, there are some uncertainties regarding the accuracy of SpHb level measurement using this technology and whether sufficient blood flow is required for reliable measurement in elderly people. If the Hb measurement using Rad-67^TM^ can be applied to elderly people, it may be possible to measure the Hb of patients by medical professionals who are not qualified to draw blood, which would be clinically significant. This study aimed to clarify whether transcutaneous hemoglobin (SpHb) measurement using Rad-67^TM^ in elderly people is as useful as blood sampling and whether it has the ability to discriminate suspected anemia.

Methods

The study participants were community-dwelling elderly people aged 65 or older who participated in a survey on frailty. The assessment included measurement of hemoglobin (Hb) level using blood sampling, SpHb level measured through the finger using Rad-67^TM^, room temperature, and temperature and perfusion index of the finger. Statistical analyses for each measurement outcome included (1) comparisons between males and females and across measurement conditions, (2) calculation of the correlation coefficient between Hb levels using blood sampling and SpHb levels, and (3) calculation of the cut-off value for SpHb levels using the area under the receiver operating characteristic curve and Youden’s index.

Results

The SpHb level using Rad-67^TM^ was not significantly different from the Hb level using blood sampling. When the effects of room temperature, finger temperature, and perfusion index were excluded, a positive correlation was observed with the Hb level using blood sampling overall in men and in women when analyzed separately. Furthermore, the results showed that SpHb measurements may have a moderate ability to significantly discriminate between those with suspected anemia in men and in women when analyzed separately. The cut-off values of SpHb for screening for suspected anemia were < 13.5 g/dL in men and < 13.0- < 13.1 g/dL in women.

Conclusion

SpHb measurement using Rad-67^TM^ is relatively useful for understanding trends in Hb levels in elderly people and may be useful for screening for suspected anemia, using a cut-off value for SpHb measurement.

## Introduction

Anemia is a condition in which the body does not have sufficient red blood cells, which can result from several causes, including iron deficiency [1]. Red blood cells contain hemoglobin (Hb), a protein that carries oxygen [1,2]. In other words, if red blood cells are insufficient in the body, the Hb level will also be low, and the body’s ability to transport oxygen will be reduced. Particularly, anemia and reduced Hb levels in elderly people are associated with frailty and sarcopenia [3-8]. In elderly people, anemia and decreased Hb levels can be caused by several factors (e.g., loss of appetite leading to deficiencies in nutrients, such as iron, gastrointestinal bleeding, decreased synthesis of erythropoietin, decreased sex hormone levels, increased cachexia due to sarcopenia, and hematopoiesis accompanied by somatic cell abnormalities) [9].

Anemia and reduced Hb levels can cause shortness of breath even with light activity or at rest and dizziness while standing up, which can affect activities of daily living [1]. Therefore, measures to prevent anemia and maintain Hb levels in elderly people are important factors that can lead to independent daily lives. However, the required blood assessments in health checkups for elderly people in Japan are lipids, liver function, and blood glucose [10,11] and do not include red blood cell count or Hb content to evaluate anemia. For most parties, anemia-related measurements are performed using invasive methods, such as blood sampling, and are often only taken after a patient feels unwell and visits a medical institution. Although the evaluation of anemia in elderly people is important, it is currently not feasible to perform blood sampling routinely and frequently.

However, recently, simpler measurement methods, such as HemoCue^®^ (HemoCue Hb 201+ System, Ängelholm, Sweden) [12], which is invasive but can be measured by drawing a single drop of blood, and Rad-67^TM^ (Masimo Corp., Irvine, CA) [13], which can measure Hb levels noninvasively using a sensor that measures absorbance, such as an SpO_2_ meter, have been reported. The Rad-67^TM^ consists of a portable tablet-type main unit and a sensor and can measure transcutaneous Hb levels (SpHb^®^, hereafter SpHb) from the finger [13]. The usefulness of this device has been partially proven in children, perioperative patients, and pregnant women, as a positive correlation has been observed between Hb levels using blood sampling and SpHb levels [14-18].

However, elderly people significantly differ from children, perioperative patients, and pregnant women in that the number of blood vessels in the elderly decreases with age, and blood flow in the deep layers of the skin tends to decrease [19]. Therefore, there are some uncertainties regarding the accuracy of SpHb level measurement using Rad-67^TM^ and whether the blood flow in elderly people is sufficient for accurate measurement. If the Hb measurement using Rad-67^TM^ is also useful in elderly people, it may be possible for patients and medical professionals who are unable to draw blood to measure Hb, which would be clinically significant.

Therefore, this study aimed to clarify whether SpHb measurement using Rad-67^TM^ in elderly people is as useful as blood sampling and whether it has the ability to discriminate suspected anemia.

## Materials and methods

Study population

**Figure 1:**
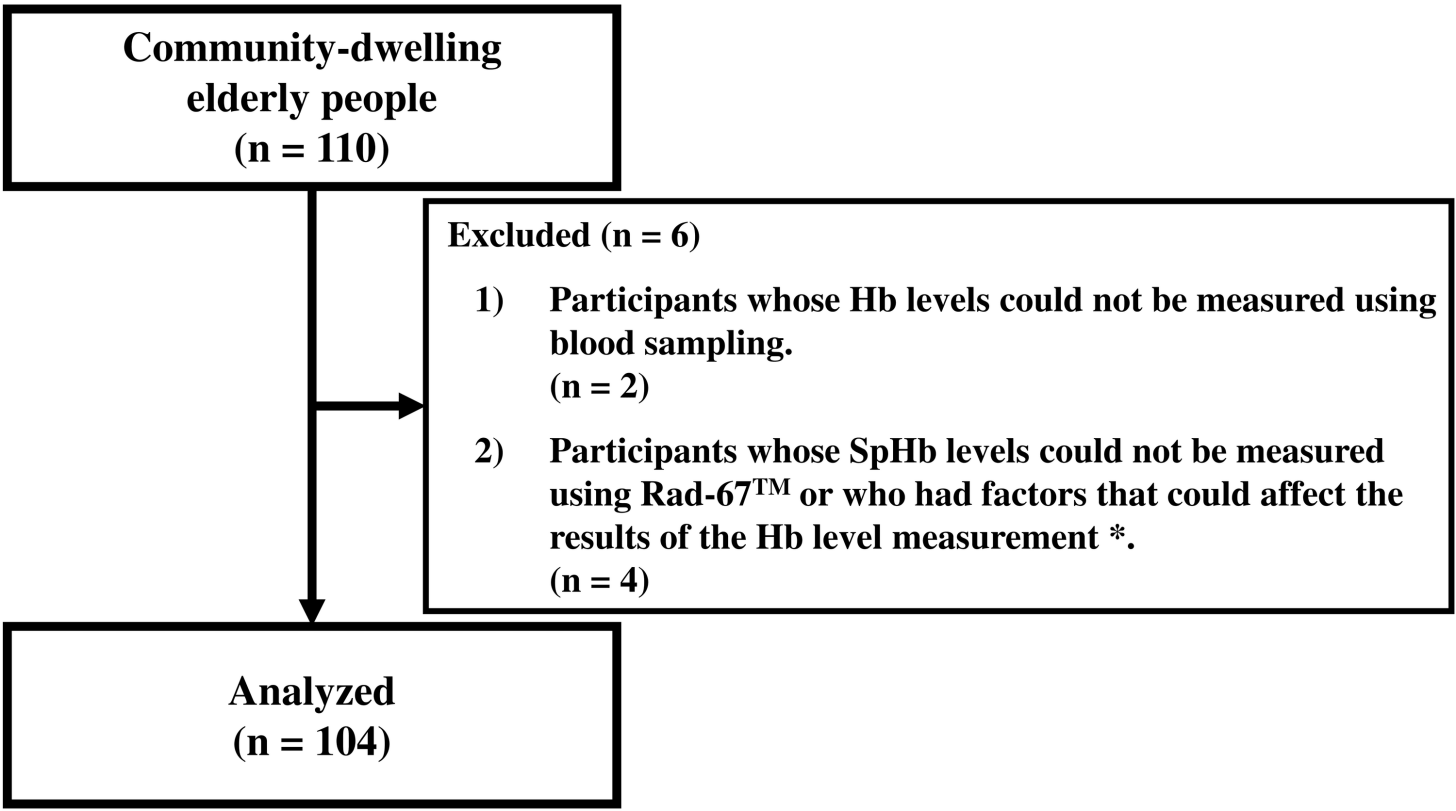
Study flow diagram ^*^e.g., Participant applying nail polish. Participants who had been diagnosed with methemoglobinemia, hemoglobinopathies, or hemoglobin synthesis disorders.

The study participants were 110 community-dwelling elderly people aged 65 or older who understood the content of the study and expressed their consent in writing signature, and participated in a survey on frailty at the Mihara campus of the Prefectural University of Hiroshima from February 1 to 4, 2025 (Figure 1).

Participants whose Hb levels could not be measured using blood sampling, those whose SpHb levels could not be measured using Rad-67 ^TM^ , or those who had factors that could affect the results of the Hb level measurement (e.g., participant applying nail polish, participants who had been diagnosed with methemoglobinemia, hemoglobinopathies, or Hb synthesis disorders) were excluded from this study. The final analysis included data from 104 participants.

Ethics statement

This study was conducted in accordance with the research ethics regulations of the Prefectural University of Hiroshima and the Declaration of Helsinki. Informed consent was obtained from the participants in writing through an explanatory paper, along with a verbal explanation. They were informed that there would be no disadvantages if they did not provide consent. This study was approved by the Research Ethics Committee of the Prefectural University of Hiroshima (Approval No.: 24MH023).

Hemoglobin measurement

Hb levels were measured using the following two methods: (1) invasive method: a doctor or nurse collected blood samples and sent them to an external clinical laboratory for analysis (Hb-Lab), and (2) noninvasive method: transcutaneous Hb (SpHb) levels were measured using an Hb measuring device (Rad-67 ^TM^ ).

In Method 1, 2 mL of collected blood was treated with a reagent for use with the multiparameter automated blood cell analyzer only (Sysmex Corporation, Hyogo, Japan), and Hb level was analyzed using a multiparameter automated blood cell analyzer, XR-1000 (Sysmex Corporation, Hyogo, Japan) or XN-1000 (Sysmex Corporation, Hyogo, Japan). Based on this Hb-Lab value, men with an Hb-Lab value < 13.0 g/dL and women with an Hb-Lab value < 12.0 g/dL were suspected of anemia, whereas men with an Hb-Lab value ≥ 13.0 g/dL and women with an Hb-Lab value ≥ 12.0 g/dL were not suspected of anemia, based on the World Health Organization (WHO) definition [20].

In Method 2, the device used for SpHb measurement was Rad-67^TM^ (Masimo Corp.), and its measurement sensor was a rainbow DCI-mini sensor (Masimo Corp.). According to the operator’s manual of Rad-67^TM^ [21], SpHb was measured using the Rad-67^TM^ and rainbow DCI-mini sensors by passing light from various types of light-emitting diodes (LEDs) through the living body and analyzing the signal that reaches the photodiode (light-receiving part), taking advantage of the fact that oxygenated Hb, deoxygenated Hb, and plasma have properties that easily absorb light of different specific wavelengths. The maximum luminance of the strongest light was ≤ 25 mW (rated). After receiving the signal from the sensor, the Rad-67^TM^ used a proprietary algorithm to calculate the SpHb as the total Hb level. In principle, the measurement site was the little finger of the dominant hand. However, if this was difficult, other fingers were used. Based on previous studies [15], measurements were generally conducted twice, with a third measurement being conducted if the difference between the first and second measurements was ≥ ±1.0 g/dL. For minimizing measurement error, participants placed their hands on the table and tried to minimize body movement. The Hb measurements were performed at the recommended room temperature of 0-35°C [21]. ​​​​​​​​​​​​​​The measurements in this study were the first measurement (First), the average of the first and second measurements (Ave. 1), and the average of the first to third measurements (Ave. 2).

Environment of measurement

To examine the influence of the measurement environment, the room temperature and the temperature of the finger to be measured for SpHb were measured immediately before the first SpHb measurement. In addition, the perfusion index was recorded simultaneously with SpHb at each measurement time using the Rad-67 ^TM^ and rainbow DCI-mini sensors. The perfusion index is calculated from the ratio of pulsatile to nonpulsatile signals in peripheral tissues and indicates peripheral perfusion status [21].

Statistical analyses

The effect size was determined to be the correlation coefficient r = 0.548 between Hb-Lab and SpHb in a previous pediatric study [15], with a statistical power of 0.8 and α = 0.05, and the minimum sample size was 21 patients. This study included 23 males and 81 females, indicating that the sample size was appropriate for both sexes. Statistical processing was performed to confirm sex differences in the characteristics of the participants, such as age, Hb-Lab level, and measurement environment. First, we used the Shapiro-Wilk test to check for normality; if normality was found, we performed a t-test, and if normality was not found, we performed the Mann-Whitney U test. To compare the Hb levels under each measurement condition (SpHb (First), SpHb (Ave. 1), SpHb (Ave. 2)), we used the Shapiro-Wilk test to check for normality and then performed a repeated one-way analysis of variance (ANOVA) if normality was found and a Friedman test if normality was not found. For post-hoc testing, Dunnett’s test was performed using Hb-Lab for comparison. Furthermore, to examine the differences between Hb-Lab and SpHb levels in each measurement condition, we checked for normality using the Shapiro-Wilk test and then performed a repeated one-way ANOVA if normality was found and a Friedman test if normality was not found.

Spearman’s test was performed to examine the correlation between Hb-Lab and SpHb levels in each measurement condition. In addition, to examine the correlation between Hb-Lab and SpHb levels in each measurement condition after excluding the effects of room temperature at the time of measurement, the temperature of the finger being measured, and the perfusion index, we performed a partial correlation analysis in which the effects of room temperature at the time of measurement, temperature of the finger being measured, and perfusion index were controlled variables.

Statistical analyses other than checking sex differences, such as age, Hb-Lab level, and measurement environment, were performed separately for all participants (total), male participants only, and female participants only. In the statistical analysis of SpHb, the ability of Hb-Lab values to discriminate between suspected and non-suspected anemia was examined using the area under the receiver operating characteristic (ROC) curve (AUC), and Youden’s index was calculated to maximize sensitivity and specificity. Statistical analyses were performed using IBM SPSS Statistics for Windows, version 29.0.2.0 (Released 2023; IBM Corp., Armonk, New York, United States), with a significance level of p < 0.05.

## Results

Characteristic outcomes

The participant characteristics are presented in Table 1. There were no significant sex differences in age or the measurement environment (room temperature, finger temperature, and perfusion index). Hb-Lab levels in female participants were significantly lower than those in male participants.

**Table 1:** Characteristics of the participants The “First” denotes the first measurement. The “Ave. 1” denotes the average calculated by first and second measurements. The “Ave. 2” denotes the average calculated by first, second, and third measurements. ^a^Statistical analysis comparing male participants and female participants was performed using the Mann–Whitney U test. ^b^Statistical analysis comparing male and female participants was performed using the t-test. ^c^The cut-off values of Hb level for suspected anemia are < 13.0 g/dL for male aged 15–65 years participants and < 12.0 g/dL for female aged 15–65 years participants according to the World Health Organization (WHO) definition [20].

Parameter	Total	Male	Female	P-value
Sex (n)	104	23	81	ｰ
Age (year)^a^	75.28 ± 4.76	76.13 ± 5.33	75.04 ± 4.59	0.487
Hb-Lab (g/dL)^b^	13.22 ± 1.05	13.79 ± 1.10	13.06 ± 0.98	0.003
Room temperature (℃)^a^	20.19 ± 1.47	19.95 ± 1.46	20.25 ± 1.47	0.288
Finger temperature (℃)^b^	24.59 ± 2.61	23.67 ± 2.41	24.85 ± 2.62	0.056
Perfusion index (First)^a^	4.09 ± 3.05	3.01 ± 2.27	4.39 ± 3.19	0.072
Perfusion index (Ave. 1)^a^	4.43 ± 3.33	3.19 ± 2.35	4.78 ± 3.49	0.062
Perfusion index (Ave. 2)^a^	4.46 ± 3.32	3.23 ± 2.42	4.81 ± 3.47	0.054
Less than cut-off value; n/n (%)^c^	22/104 (21.15)	6/23 (26.09)	16/81 (19.75)	ｰ
Third measurement performed; n/n (%)	18/104 (17.31)	4/23 (17.39)	14/81 (17.28)	ｰ

Comparison of Hb-Lab and SpHb levels

The results of the comparison of Hb-Lab with SpHb levels under each measurement condition (SpHb (First), SpHb (Ave. 1), SpHb (Ave. 2)) are shown in Table 2. There were no significant differences between Hb-Lab and SpHb levels across measurement conditions in all participants, male participants only, and female participants only. However, when post-hoc tests were performed, SpHb levels (First) in male participants showed significantly lower values than Hb-Lab levels.

**Table 2:** The comparison of Hb-Lab and SpHb levels Data are shown as averages ± standard deviations. The “First” denotes the first measurement. The “Ave. 1” denotes the average calculated by first and second measurements. The “Ave. 2” denotes the average calculated by first, second, and third measurements. ^a^Statistical analysis was performed by repeated one-way analysis of variance. ^*^p < 0.05 (Dunnett test, vs. Hb-Lab)

Parameter	Hb-Lab (g/dL)	SpHb (g/dL)			P-value
		First	Ave. 1	Ave. 2	
Total (n = 104)^a^	13.22 ± 1.05	13.19 ± 1.16	13.18 ± 0.99	13.19 ± 0.96	0.811
Male (n = 23)^a^	13.79 ± 1.10	13.32 ± 1.00^*^	13.41 ± 1.00	13.43 ± 0.98	0.091
Female (n = 81)^a^	13.06 ± 0.98	13.15 ± 1.20	13.11 ± 0.98	13.12 ± 0.95	0.606

Comparison of the difference between SpHb and Hb-Lab levels

The results of the differences between SpHb levels in each measurement condition (SpHb (First), SpHb (Ave. 1), SpHb (Ave. 2)) and Hb-Lab levels are shown in Table 3. No significant differences were observed between SpHb levels across measurement conditions and Hb-Lab levels in all participants, male participants only, and female participants only.

**Table 3:** The comparison of difference of SpHb and Hb-Lab levels Data are shown as averages ± standard deviations. The “First” denotes the first measurement. The “Ave. 1” denotes the average calculated by first and second measurements. The “Ave. 2” denotes the average calculated by first, second, and third measurements. ^a^Statistical analysis was performed using repeated one-way analysis of variance. ^b^Statistical analysis was performed using the Friedman test.

Parameter	Difference (SpHb - Hb-Lab)			P-value
	First	Ave. 1	Ave. 2	
Total (n = 104)^b^	-0.03 ± 1.15	-0.04 ± 1.01	-0.03 ± 0.99	0.701
Male (n = 23)^a^	-0.47 ± 1.15	-0.38 ± 1.11	-0.36 ± 1.08	0.157
Female (n = 81)^a^	0.09 ± 1.13	0.05 ± 0.96	0.06 ± 0.94	0.575

Correlation analysis between Hb-Lab and SpHb levels

**Figure 2:**
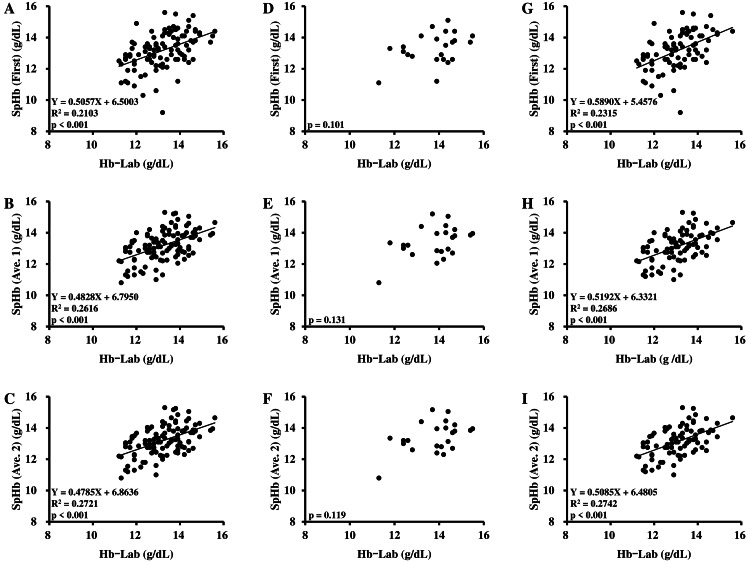
Correlation chart between Hb-Lab and SpHb levels A–C: Results of all participants; D–F: Results of male participants; G–I: Results of female participants. The “First” denotes the first measurement. The “Ave. 1” denotes the average calculated by first and second measurements. The “Ave. 2” denotes the average calculated by first, second, and third measurements.

The correlations between Hb-Lab and SpHb levels in each measurement condition (SpHb (First), SpHb (Ave. 1), SpHb (Ave. 2)) in all participants, male participants only, and female participants only are shown in Table 4 and Figure 2. A significant positive correlation was observed between Hb-Lab and SpHb levels across measurement conditions in all participants and in female participants only. No significant correlation was observed between Hb-Lab and SpHb levels across measurement conditions in male participants only.

Furthermore, when the effects of room temperature, finger temperature, and perfusion index were excluded, a significantly positive correlation was found between Hb-Lab and SpHb levels across measurement conditions in all participants, male participants only, and female participants only.

**Table 4:** The results of correlation analysis between Hb-Lab and SpHb levels The “First” denotes the first measurement. The “Ave. 1” denotes the average calculated by first and second measurements. The “Ave. 2” denotes the average calculated by first, second, and third measurements.

Variable 1	Variable 2	Control variable	Total (n = 104)		Male (n = 23)		Female (n = 81)	
			Correlation coefficient	P-value	Correlation coefficient	P-value	Correlation coefficient	P-value
Hb-Lab	SpHb (First)	ｰ	0.479	< 0.001	0.351	0.101	0.519	< 0.001
	SpHb (Ave. 1)	ｰ	0.492	< 0.001	0.324	0.131	0.517	< 0.001
	SpHb (Ave. 2)	ｰ	0.502	< 0.001	0.334	0.119	0.519	< 0.001
Hb-Lab	SpHb (First)	Room temperature	0.440	< 0.001	0.424	0.049	0.463	< 0.001
		Finger temperature	0.495	< 0.001	0.572	0.005	0.492	< 0.001
		Perfusion index	0.523	< 0.001	0.462	0.030	0.533	< 0.001
	SpHb (Ave. 1)	Room temperature	0.501	< 0.001	0.468	0.028	0.506	< 0.001
		Finger temperature	0.563	< 0.001	0.632	0.002	0.542	< 0.001
		Perfusion index	0.597	< 0.001	0.527	0.012	0.593	< 0.001
	SpHb (Ave. 2)	Room temperature	0.512	< 0.001	0.488	0.021	0.512	< 0.001
		Finger temperature	0.571	< 0.001	0.652	0.001	0.546	< 0.001
		Perfusion index	0.609	< 0.001	0.545	0.009	0.601	< 0.001

Analysis of the ROC curve of SpHb for suspected anemia

**Figure 3:**
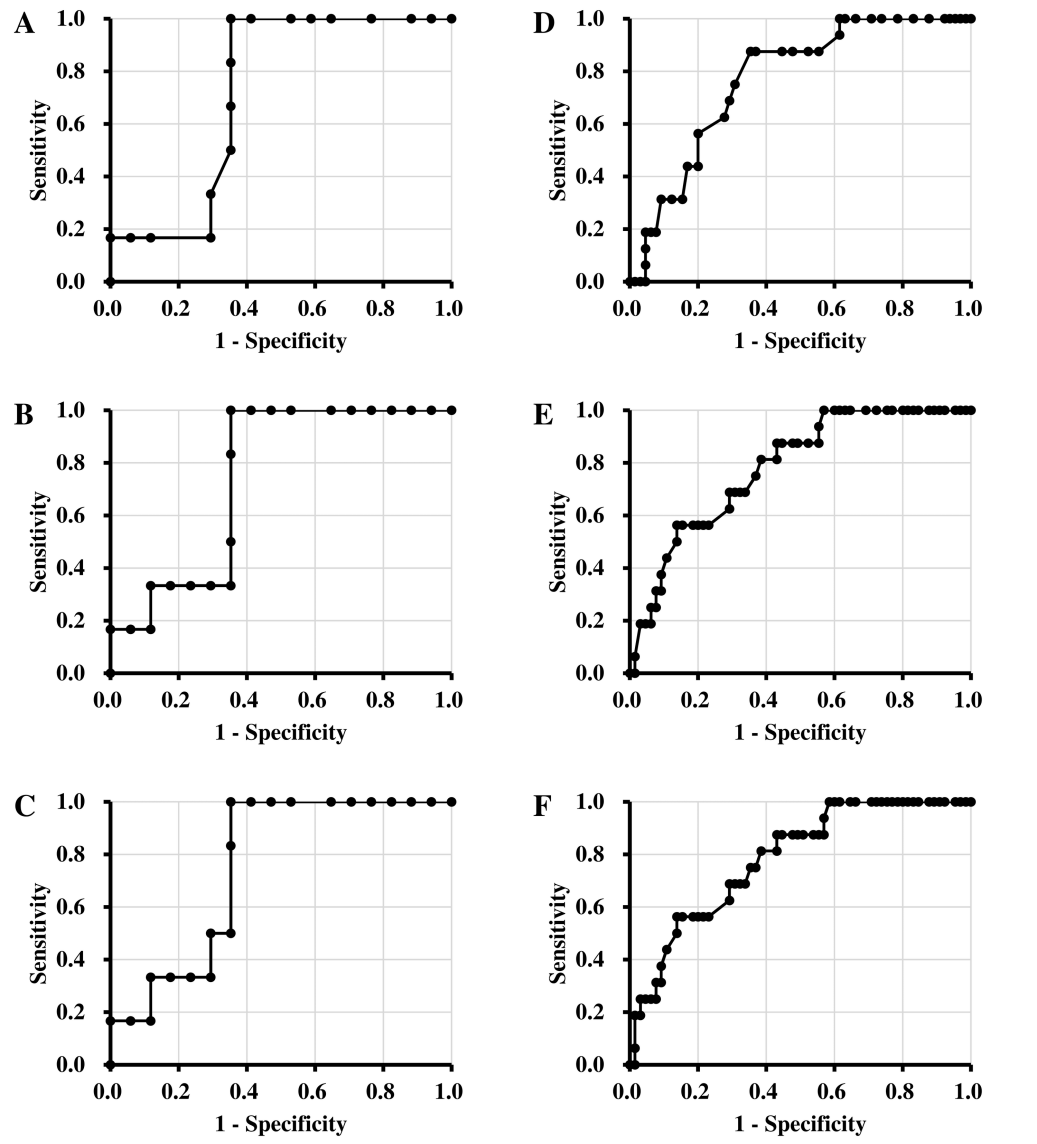
The ROC curve A–C: Results for male participants. D–F: Results for female participants. A, D: ROC curve calculated by Hb-Lab and SpHb of first measurement. B, E: ROC curve calculated by Hb-Lab and SpHb of first and second measurements. C, F: ROC curve calculated by first, second, and third measurements.

The results of the ROC curve analysis are presented in Figure 3 and Tables 5-6. The AUC in male participants was 0.721-0.755 under each measurement condition, with significant differences observed, and the value increased with the number of measurements. The cut-off value for suspected anemia in male participants using SpHb measurement based on Youden’s index was < 13.5 g/dL under all measurement conditions. The AUC in female participants was 0.768-0.784 under each measurement condition, with significant differences observed, and the value increased with the number of measurements. The cut-off values for suspected anemia in female participants using SpHb measurement based on Youden’s index were < 13.0 under single measurement (First), and < 13.1 g/dL under multiple measurement (Ave. 1 and Ave. 2).

**Table 5:** The analysis of ROC curve and cut-off value for suspected anemia The “First” denotes the first measurement. The “Ave. 1” denotes the average calculated by the first and second measurements. The “Ave. 2” denotes the average calculated by the first, second, and third measurements. ROC: receiver operating characteristic

Sex	Variable	Area under the ROC curve (AUC)	P value	95% CI	Cut-off (g/dL)
Male (n = 23)	SpHb (First)	0.721	0.041	0.509 – 0.932	< 13.5
	SpHb (Ave. 1)	0.745	0.019	0.539 – 0.951	< 13.5
	SpHb (Ave. 2)	0.755	0.012	0.555 – 0.955	< 13.5
Female (n= 81)	SpHb (First)	0.768	< 0.001	0.657 – 0.879	< 13.0
	SpHb (Ave. 1)	0.782	< 0.001	0.671 – 0.893	< 13.1
	SpHb (Ave. 2)	0.784	< 0.001	0.671 – 0.896	< 13.1

**Table 6:** Accuracy of each cut-off value for suspected anemia The “First” denotes the first measurement. The “Ave. 1” denotes the average calculated by the first and second measurements. The “Ave. 2” denotes the average calculated by the first, second, and third measurements. PPV, positive predictive value; NPV, negative predictive value. ^a^The cut-off value of Hb-Lab for suspected anemia ^b^The cut-off value of SpHb (First) for suspected anemia ^c^The cut-off value of SpHb (Ave. 1) for suspected anemia ^d^The cut-off value of SpHb (Ave. 2) for suspected anemia

Sex	Variable	Cut-off (g/dL)	Sensitivity (%)	Specificity (%)	PPV (%)	NPV (%)
Male (n = 23)	SpHb (First)	< 13.0^a^	50.0	64.7	33.3	78.6
		< 13.5^b^	100.0	64.7	50.0	100.0
	SpHb (Ave. 1)	< 13.0^a^	33.3	64.7	25.0	73.3
		< 13.5^c^	100.0	64.7	50.0	100.0
	SpHb (Ave. 2)	< 13.0^a^	33.3	70.6	28.6	75.0
		< 13.5^d^	100.0	64.7	50.0	100.0
Female (n = 81)	SpHb (First)	< 12.0^a^	31.3	90.8	45.5	84.3
		< 13.0^b^	87.5	64.6	37.8	95.5
	SpHb (Ave. 1)	< 12.0^a^	31.3	90.8	45.5	84.3
		< 13.1^c^	81.3	61.5	34.2	93.0
	SpHb (Ave. 2)	< 12.0^a^	31.3	92.3	50.0	84.5
		< 13.1^d^	81.3	61.5	34.2	93.0

## Discussion

The noninvasive SpHb measurements using Rad-67^TM^ showed no significant differences from the invasive Hb measurements using blood sampling in the total participants, in male participants only, and in female participants only, and a positive correlation was observed. These results suggest that SpHb measurements are as useful as Hb measurements using blood samples from elderly people. These results are similar to those of previous studies on children, perioperative patients, and pregnant women [14-18]. However, it was assumed that SpHb measurement in males may occur due to measurement errors in a single measurement and may be strongly influenced by factors, such as room temperature, finger temperature, and perfusion index.

The principle of SpHb measurement using Rad-67^TM^ is to pass light from various types of LEDs through the blood at the irradiation site and analyze the signal that reaches the photodiode (light-receiving part) [21]. In other words, SpHb measurement may depend on the blood flow at the fingertip. The human body uses various mechanisms to regulate temperature. In the skin, blood vessels in the dermis constrict when exposed to a cold environment, reducing blood flow and inhibiting heat loss, and dilate when exposed to a hot environment, increasing blood flow and promoting heat loss [2,22]. In this study, the average room temperature at the time of SpHb measurement across total participants was 20.19 ± 1.47°C. The average temperature in the city where the experiment was conducted was 1.93 ± 2.85 °C over the four measurement days [23]. Therefore, it was assumed that the participants were exposed to a cold environment, which reduced the blood flow to the skin of their fingertips to suppress heat loss and lower the measured finger temperature. Consequently, the perfusion index decreased and SpHb measurement from the blood flow in the blood vessels of the contracted finger was hindered, which may have influenced the SpHb measurement results. Elderly people have a lower ability than younger people to maintain their body temperature when exposed to cold environments [24]. In addition, the body temperature in an elderly person is influenced by sex [24], and the body temperature of male participants, including the elderly, is lower than that of female participants [25]. This suggests that males may have a lower ability to regulate body temperature in cold environments than females. In this study, finger temperatures and perfusion index tended to be lower in male participants than in female participants, although the difference was not significant. In this study, if we assume that the male participants had a lower ability to regulate their body temperature than the female participants, it is possible that the male participants had an excessive defensive reaction to a cold environment, that is, dermal vasoconstriction. Consequently, it is thought that SpHb measurement is strongly affected by a decrease in blood flow at the fingertips in males.

Rad-67^TM^ has been shown to be positively correlated with Hb levels measured using blood sampling [14-18]. As mentioned in the Introduction section, anemia is a condition in which the body does not have sufficient red blood cells [1], and red blood cells contain the protein Hb, which carries oxygen [1,2]. Therefore, it may be possible to infer suspected anemia by measuring Hb levels. However, the accuracy of Hb measurement using Rad-67^TM^ is not suitable for discriminating anemia [14,17,18]. Saengnipanthkul et al. [14] compared Hb measurements using blood sampling with SpHb measurements using Rad-67^TM^ to screen for anemia in healthy infants (Hb < 11.0 g/dL). The results suggest that although a positive correlation was observed between the two measurement methods, SpHb values may be falsely detected as high, especially in infants with anemia. Koech et al. [17] compared Hb measurements using blood sampling with SpHb measurements using Rad-67^TM^ for detecting anemia in pregnant women (first trimester, Hb < 11.0 g/dL; second trimester, Hb < 10.5 g/dL; third trimester, Hb < 11.0 g/dL). The results showed that, although a positive correlation was found between the two measurement methods, many women with anemia were misclassified as not having anemia based on their SpHb results. Ke et al. [18] compared Hb measurements using blood sampling with SpHb measurement using Rad-67^TM^ for the detection of anemia in preoperative patients (Hb < 13.0 g/dL). Although a positive correlation was found between the two measurement methods, it was insufficient for estimating actual Hb levels, and the sensitivity for detecting preoperative anemia was low, especially in females. However, in this study, the AUC was used to determine the cut-off value for suspected anemia based on Youden’s index. The results were as follows: AUC = 0.721-0.755 (cut-off < 13.5 g/dL) for elderly male participants (sensitivity, 100%; significant difference) and AUC = 0.768-0.784 (cut-off < 13.0-< 13.1 g/dL) for elderly female participants (sensitivity, 81.3-87.5%; significant difference), suggesting that SpHb measurement using Rad-67^TM^ may have a significant moderate ability to discriminate suspected anemia in elderly male and female participants. The WHO definition of suspected anemia does not include elderly people aged 66 years and above [20]. If we assume that the same WHO definition for suspected anemia can be applied to people aged 66 years and above as those aged 15-65 years, the results of this study suggest that noninvasive SpHb measurement using Rad-67^TM^ is useful for understanding trends in Hb levels in elderly people and that using a cut-off value for SpHb measurement may be useful for screening for suspected anemia. Previous studies that examined the ability of Rad-67^TM^ to discriminate anemia [14,17,18] cannot be strictly compared with this study because the characteristics of the participants and the Hb level criteria used to discriminate the presence or absence of anemia were different. However, the results of this study differ greatly from those of previous studies in that they may discern suspected anemia in elderly participants. The lifespan of red blood cells is approximately 90-120 days [1,2]. Therefore, even when red blood cell counts and Hb levels are measured during health checkups in Japan, as mentioned in the Introduction section, the frequency of Hb measurements is low. The results of this study suggest that high-frequency noninvasive measurement of Hb levels may be useful for the early detection of suspected anemia in elderly people and for preventing frailty in the elderly.

This study has some limitations. The participants were limited to elderly people with sufficient mobility to participate in the survey of frailty survey, the experimental environment may have been affected by the season (outdoor temperature), the small number of male participants may have influenced the results in male, and anemia should be diagnosed by a physician based on a blood test and physical examination [1]. Thus, SpHb measurement using the Rad-67^TM^ cannot be used to diagnose anemia.

Furthermore, this was a highly health-conscious group that chose to participate in the frailty survey and was different from that of facility residents. Therefore, the possibility that the participants of this study had a high blood flow to the fingertips daily is indisputable. It is considered that we must assess with careful the values of SpHb measured from elderly men and elderly people who in low health-conscious group without enough blood flow in their fingertips, and in some situations, it may be necessary to compare the SpHb and Hb from blood sampling. In future studies, it will be necessary to eliminate the influence of mobility ability, other conditions of participants, and outdoor temperature and to increase the number of male participants.

## Conclusions

In this study, the noninvasive measurement of Hb levels using Rad-67^TM^ was positively correlated with the invasive measurement of Hb levels using blood sampling in elderly people. Therefore, the noninvasive measurement of Hb levels using Rad-67^TM^ is sufficiently useful for understanding trends in Hb levels in elderly people. In male and female participants, it was hypothesized that using a cut-off value for SpHb measurement may be useful for screening to visit the hospital for suspected anemia. These results indicate that it is possible to measure Hb levels frequently to effectively assess the state of oxygen transport capacity in elderly people, which is of great clinical significance. Furthermore, the patients and medical professionals can measure the SpHb, because it does not require blood sampling. Therefore, it also considered that measurement of Hb levels using Rad-67^TM^ is useful in that it can provide instant results in a wide range of situations. However, this study did not investigate that effects of mobility of elderly people and season, and further studies are required.
